# Optimal spare parts management for vessel maintenance scheduling

**DOI:** 10.1007/s10479-018-2907-y

**Published:** 2018-06-01

**Authors:** Ramez Kian, Tolga Bektaş, Djamila Ouelhadj

**Affiliations:** 1 Department of Industrial Engineering, University of Tabriz, Tabriz, Iran; 2 Centre for Operational Research Management Science and Information Systems (CORMSIS), Southampton Business School, University of Southampton, Highfield, Southampton, SO17 1BJ UK; 3 Department of Mathematics, Centre for Operational Research and Logistics (CORL), University of Portsmouth, Portsmouth, PO1 3HF UK

**Keywords:** Spare part management, Maritime logistics, Shortest path, Dynamic programming, Integer programming

## Abstract

Condition-based monitoring is used as part of predictive maintenance to collect real-time information on the healthy status of a vessel engine, which allows for a more accurate estimation of the remaining life of an engine or its parts, as well as providing a warning for a potential failure of an engine part. An engine failure results in delays and down-times in the voyage of a vessel, which translates into additional cost and penalties. This paper studies a spare part management problem for maintenance scheduling of a vessel operating on a given route that is defined by a sequence of port visits. When a warning on part failure is received, the problem decides when and to which port each part should be ordered, where the latter is also the location at which the maintenance operation would be performed. The paper describes a mathematical programming model of the problem, as well as a shortest path dynamic programming formulation for a single part which solves the problem in polynomial time complexity. Simulation results are presented in which the models are tested under different scenarios.

## Introduction

Spare part management deals with the procurement and ordering of the components of equipments used in manufacturing or service industries, in order to keep equipment in operating condition (Kennedy et al. 2002). The treatment of spare parts in most manufacturing systems is different to that in traditional inventory management as they are neither intermediate nor final products to be sold to end users. In particular, the insufficiency or unavailability of spare parts can lead to long machine downtimes or disruptions in the relevant production or service system. Moreover, storing extra spares may incur significant carrying costs, their degradation, or other warehousing issues. In addition, demand prediction in spare part management is more difficult than that of finished products. If classical forecasting methods are used without taking into account the information about the condition of the system in which spare parts are used, it will be difficult to produce a high quality demand forecast.

Any spare part being used in a given operation follows a two-stage process before breakdown. The first stage is the time interval between the start time of the operation to the identification of a fault, and the second stage is from fault identification to failure time. The latter is sometimes referred to as the failure delay-time, and denoted the P-F interval (Wang et al. 2009).

A good spare parts policy should prescribe an action so as to be able to deal with a potential failure of the operation, and depends on the condition of the equipment used and the maintenance policy used (Ilgin and Tunali 2007). A maintenance action can typically be classified as corrective maintenance and preventive maintenance. Furthermore, preventive maintenance can be time-based maintenance, condition-based maintenance and predictive maintenance. In the corrective policy, the equipment is used until failure, and is either repaired or replaced once it breaks down. The time-based preventive maintenance policy corresponds to a periodic review of the condition of the equipment, at which point a repair or a maintenance action is decided on, as appropriate. In predictive maintenance, the condition of the equipment is continuously monitored using real-time information from sensors that monitor the status of the equipment, and replacement or repair decisions are made accordingly. In preventive maintenance, if an inspection is carried out during the delay-time, the fault may be detected and repaired. In contrast, the predictive policy requires that the failures are detected at the earliest possible time, which allows more time for reaction. With the availability of Condition-Based Monitoring (CBM) systems, inventory levels are lower as the procurement process is triggered only by the identification of a potential failure, and if the delay-time is longer than the part lead-time, then there is no need to stock a spare.

Recent progress and developments in monitoring devices have increased their take-up in various industries, resulting in a shift from preventive to predictive maintenance policies. An application in the auto-mobile industry, for example, has seen the use of condition-monitoring tools, which analyze chemical compounds in engine oil, and if necessary, provide an alert to indicate the need for oil replacement. This is in contrast to the more traditional means of monitoring based on, for example, vehicle mileage (see Rigol 2011).

A more relevant industry where the use of CBM is expected to bring significant benefits is maritime logistics. According to the Review of Maritime Transport Report (UNCTAD, 2015), the global seaborne shipments have increased by 3.4% in 2014, while world gross domestic product (GDP) increased marginally by 2.5% at the same year. One significant source of delay within the sector is unforeseen breakdowns in the hauling system of the vessels (e.g., the engine), which potentially result in long service times and deviating from the schedule on which the vessel is expected to operate, implying financial penalties. Ship owners tend to outsource most of their maintenance and procurement activities to third parties, who provide them with a more flexible maintenance environment and spare part procurement. Whilst commercial ships undergo maintenance as part of mandatory surveys every 2.5 years on average (Eruguz et al. 2017) where major repair operations are carried out, the responsibility for maintenance between the periodic surveys lies with the owner. For unpredictable activities, employing a CBM system for critical parts of the engine will provide a platform that allows benefiting from advance information in maintenance scheduling and spare part management.

Although spare part management and CBM control activities have previously and individually been studied, their integration with logistics management to improve the ordering policy and maintenance scheduling has not yet been given full attention in the literature. The present paper aims to contribute to this area of research. In particular, we study a spare part ordering and maintenance scheduling problem arising in the maritime sector, where a CBM system is used to monitor the condition of a vessel engine and its parts. The particular setting assumed here is of liner shipping in cargo maritime operations, where vessels follow regular trade routes on fixed schedules that are normally designed at the tactical level of planning, e.g., three to six months (Wang et al. 2014). In this case, a vessel journey is a sequence of port visits where timetables are published that include the planned arrival and departure times to each port. The number of port calls made by an individual vessel varies with the type of cargo carried and the trade route, among other factors, but can range from direct services from one port to another to routes with significantly many calls (see, e.g., Wang and Meng 2012) for a particular route from a global liner shipping company that includes 27 ports altogether. For further details on liner shipping operations, the reader is referred to Meng et al. (2013). It is assumed that the real-time information provided by the CBM system provides an indication as to the failure time of one or several parts of the ship engine. The relevant optimization problem is to use this information to decide on the time and location of spare part orders, where the latter also corresponds to the location of the maintenance operations. The objective is to minimize the total cost that includes any penalties for delays or downtimes arising from potential failures, as well as the procurement and maintenance costs.

The rest of the paper is organized as follows: in the next section we briefly review the relevant literature. Section 3 presents a formal definition and a mathematical formulation of the problem. For a special case of the problem with a single part in Sect. 3.3, we describe a dynamic programming algorithm. Section 4 provides numerical examples and simulation analysis to compare various scenarios. Section 5 presents the conclusions.

## Overview of the relevant literature

This section presents a brief literature review on preventive and predictive maintenance scheduling. We will not attempt to review the area of maintenance and spare parts, as the literature on this topic is rich, and instead we refer the reader to the reviews by Paz and Leigh (1994) on maintenance policies, issues and techniques, by Kennedy et al. (2002) on spare part inventories, and Prajapati et al. (2012) on condition-based maintenance.

Most studies related to integrated maintenance operations and spare part management assume a *preventive* policy, and consequently aim to optimize inspection intervals and order quantities. Among these, Deris et al. (1999) model the periodic maintenance scheduling problem for a fleet of ships using constraint based reasoning and solve it using a genetic algorithm. Xie and Wang (2008) combine a continuous review ordering policy (*s*, *S*) with an inspection period for condition-based preventive maintenance. A joint optimization problem involving the order quantity, the order interval and the inspection interval for spare parts is introduced by Wang et al. (2009), who present a general approach integrating simulation with a genetic algorithm to identify a near-optimal strategy. Wang (2012) describes a joint optimization problem including both the inventory control of the spare parts and the maintenance inspection interval. The problem assumes a stochastic demand, for which the author describes a stochastic dynamic programming model for finding optimal solutions over a finite time horizon. A joint maintenance and spare parts ordering problem is introduced in Panagiotidou (2014). Finally, Gan et al. (2015) study a joint optimization problem of maintenance, buffer inventory, and the number of spare parts arising in a production system, and describe a genetic algorithm.

As for the literature on *predictive* maintenance, Sundberg (2003) discuss the opportunities of using CBM in maritime industry from a managerial standpoint. A cohesive model for managing maintenance operations and spare part inventory for a single production system is described by Rausch (2008) that uses condition based maintenance and Bayesian analysis. Elwany and Gebraeel (2008) develop a sensor-driven decision model for component replacement and spare parts inventory. They use a degradation modeling framework for computing the remaining-life distributions within the inventory decision models. Reimann et al. (2009) describe a scheduling algorithm which makes use of CBM data to determine when maintenance should be performed, and use a simulation model to compare the cost of predictive and corrective maintenance policies. Liao and Rausch (2010) address a joint production and spare part inventory control strategy driven by CBM for a piece of manufacturing equipment. They use constrained least squares approximation in conjunction with simulation-based optimization within a two-step heuristic to determine the optimal base-stock level of spare parts. Louit et al. (2011) calculate conditional reliability function based on a well-known proportional hazard model to calculate the remaining useful life for spare parts to propose condition based stock-holding decisions, where the decision variable is the time to order a spare for an equipment whose condition is being monitored. Koochaki et al. (2011) study maintenance policies at a plant-wide level and investigate the effectiveness of condition based maintenance. They develop a simulation model to explore the effects of costs and equipments availability as well as line efficiency. They show that CBM yields the best performance in loosely coupled processes, but adversely affect production line efficiency with tightly coupled processes due to the increase in the blockage of equipments. Zanjani and Nourelfath (2014) study coordinated spare part inventory and operations planning problem for a third party maintenance provider faced with strict due dates for delivering repaired equipment. They describe a mathematical programming model to minimize the procurement, inventory, and late delivery costs by finding the optimal number of maintenance jobs and the order quantities in a periodic problem. Camci (2015) discusses condition-based maintenance scheduling for geographically distributed assets, models it as a variant of a traveling repairman problem and describes a genetic algorithm.

### Contribution highlights

In this work, we study a spare part ordering problem arising within a logistics system in which the demand point (i.e., the vessel) is not stationary. This implies that the the problem parameter values depend on the position of the demand point at a given point in time. We contribute to the literature of maintenance scheduling models by (i) addressing a practical problem in the maritime sector in which maintenance policies and logistics operations are integrated, (ii) presenting mathematical programming formulations that yield optimal solutions to the problem for single and multiple parts, and (iii) quantifying the benefits of using a CBM system in terms of overall cost and in relation to system parameters including lead-time and remaining-life.

## Problem statement

The problem studied in this paper is motivated by a practical situation arising in the maritime sector and is related to spare part procurement for a vessel engine. In particular, we consider a single vessel operating on a predefined route, whose engine is equipped with CBM sensors. The sensors continuously monitor and transmit vibration data from the critical parts of the engine to a central location. We assume that the vibration data is analyzed using signal processing techniques in order to detect any malfunction, which, in turn, is used to predict the remaining-life of the part.

The vessel must complete a given route that consists of *K* segments or legs, where each leg is defined between two successive ports on the route. The vessel is also given a schedule, which prescribes the scheduled arrival time for each port that a vessel must visit. Any delay in the arrival time results in penalty costs to the ship operator. The standard transit time on each segment of the route, and the service times for loading/unloading and for ordinary maintenance at each port are assumed to be known and constant.

During the voyage on each segment of the route, if the data transmitted from the CBM system indicates that the engine part is not in a healthy condition and a potential failure is detected, it provides a warning, which triggers an action for procurement of the part. The system also prescribes the remaining-life of the part. Engine parts are usually expensive, for which reason ship owners tend to avoid stocking spare parts (e.g., in warehouses or on-board). Instead, spare parts are ordered when needed. In traditional inventory management terminology, the spare part ordering policy here is lot-for-lot for each part, and follows a $(S,S-1)$ inventory policy in which the inventory position *S* and the reorder point, $S-1$, differ only by a single unit. Such policy is generally applicable to critical parts which exhibit Poisson demand (see Louit et al. 2011).

We assume that, when a spare part is ordered, it can only be delivered to any one of the unvisited ports on the given route. Whilst, in practice, direct delivery of parts to a vessel is possible (e.g., using air transport), the cost of this operation is likely to be prohibitive, for which reason we exclude such possibilities here. Maintenance operations are usually carried out at ports after the relevant part(s) have been received. Each port has a different cost for receiving the part and for carrying out the maintenance service. We assume that each part has a lead-time that is known and constant, but differs from one port to the other given the geographically dispersed locations of the ports.

When a part is ordered for delivery to a particular port on the route, one of the two following situations arise:

1. The vessel arrives at the port before the part fails, receives maintenance at the port, and resumes the voyage as normal. The time that vessels spend at the port includes maintenance time, and any waiting for the spare part to be delivered to the port if it has not yet arrived, i.e., down-time.
2. The part fails *en-route* before the vessel reaches the designated port to which the part has been ordered, at which point a redundant system on the vessel will be activated to keep the ship moving, but at a slower sail speed.

The ship owner incurs three types of costs related to ordering and maintenance: (i) delay penalty cost due to speed reduction in the presence of failure, (ii) down-time cost which corresponds to additional time spent in a port to wait for a spare part and for maintenance, and (iii) the fixed maintenance and the procurement cost.

The spare part management and maintenance scheduling problem we model in this paper arises when a warning is issued by the CBM system, and involves, for each part that is likely to fail, deciding on the time at which the part should be ordered, and the port to which the part should be delivered. The objective is to minimize the costs associated with delay and waiting times on the port for spare part delivery, as well as those of spare part procurement and maintenance. The problem hinges on a trade-off between the cost components in deciding where to order and pick-up the spare parts. In particular, the order for the new part can be made for delivery to the first port to be visited immediately after the warning, but this may result in a large amount of down-time if the lead-time is long. Alternatively, the order can be made for delivery to one of the subsequent ports on the route, but this carries with a risk of the part failing in the meantime, as a result of which the vessel will travel at low speed until the selected port. The latter will result in delay costs. In addition, the procurement costs are different. In the light of the trade-offs, the decision of which port should be chosen for part delivery and maintenance is not obvious.

**Fig. 1:**
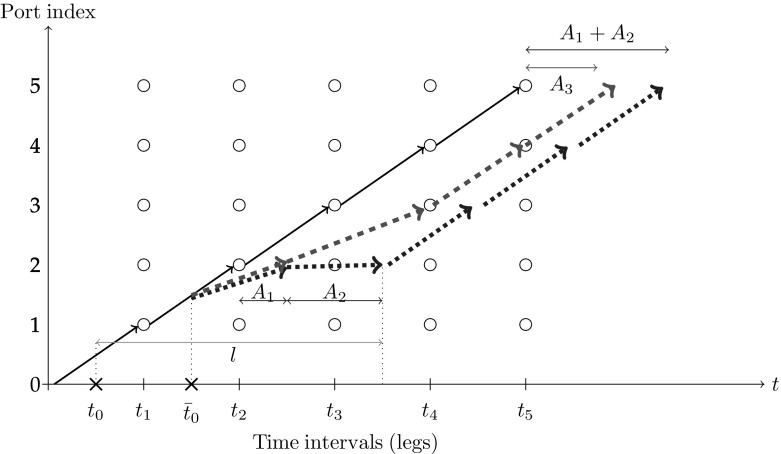
The time-space diagram of the illustrative example

Figure 1 illustrates an example of the described problem in the form of a time-space network, where a vessel is to visit five ports shown by 1, 2, 3, 4 and 5, in the given order, starting from a location indicated by 0. The times at which the vessel is scheduled to arrive at each port are shown in the horizontal axis, and the route itself is shown by the sequence of black arcs in the figure, according to which the vessel is scheduled to arrive at port 1 at time $t_1$, port 2 at time $t_2$, and so on.

The example assumes that a warning is received at time $t_0$ for a particular engine part, which also indicates the time ${\bar{t}}_0$ at which the part is due to fail. If a new part is to be ordered at time $t_0$, a decision is to be made on which port the part should be delivered to, assuming that there is *l* units of lead-time for the part from the time of placing an order. If port 2 is chosen, then the part will fail en-route, and the ship will follow the trajectory shown by the (blue) dotted arcs after time ${\bar{t}}_0$, on which it will travel at a reduced speed until port 2. The ship will then resume with a normal speed after maintenance at this port, but this is at the expense of $A_1$ units of time lost due to slower sailing speed and an additional $A_2$ units waiting for spare time to arrive at port 2. If, however, the part is ordered to be delivered at port 3, then the vessel will arrive there with slower speed along the trajectory shown by the (red) dashed arcs. As a result, it will only be delayed by $A_3$ units of time, and avoid waiting for the part as the part will already have been delivered to this port by the time the ship arrives there. The speed of the ship will revert back to normal upon leaving port 3.

As this example shows, even though a part is ordered to a later port in the sequence and this ship travels slower for a longer period of time, this may overall result in less total delay, as the red trajectory shows.

The warnings on the health status of the engine are received during the voyage at each segment of the trip. For this reason, we model and solve the problem as and when it arises, and, as a consequence, treat it within a rolling horizon framework. Such a treatment requires that optimal decisions regarding the selection of spare part pick-up or maintenance port are made after a warning has been issued by the CBM system. In other words, the problem is repeatedly solved whenever a warning is received over a leg, which we will refer to as the *phase* in which the problem is solved.

### Notation and problem modeling

We now present the formal notation that will be used to model the problem. Let ${\mathcal {P}}=\{0,\ldots ,K\}$ be the set of ports and let ${\mathcal {L}}=\{(0,1),\ldots (K-1,K)\}$ be an ordered set of route segments in which each element corresponds to the leg that connects port $(i-1) \in {\mathcal {P}}$ to port $i \in {\mathcal {P}}$. We therefore have that $|{\mathcal {L}}|=|{\mathcal {P}}|-1=K$. Let ${\mathcal {P}}'={\mathcal {P}}\backslash \{0\}$ be the index set of route segments, where the transit time on each leg $(i-1,i)$ is shown by $\tau _i$. We assume that the engine has $|{\mathcal {N}}|$ parts, where the status of each part is individually monitored. Each part has a lead-time, procurement cost and a salvage value that will be introduced in more formal terms below.

The itinerary prescribes a scheduled arrival time $t_i$ to each port $i \in {\mathcal {P}}$ and a scheduled service time $s_i$ at that port. The maintenance time is included within the berthing and service time, and is not explicitly modeled for reasons of notational simplicity. Each time unit of delay from the scheduled arrival time into port $i \in {\mathcal {P}}$ incurs a penalty cost $w_i$. Similarly, each extra time unit that the ship stays at port *i* in addition to the normal service time costs $p_i$. Any maintenance service for the part replacement at port $i \in {\mathcal {P}}$ incurs $f_i$ units of cost. Table 1 presents the notation for all the parameters used.

**Table 1:** Input parameters

Parameter	Description
*K*	Total number of legs (planning horizon)
${\mathcal {N}}$	Set $\{1,\ldots N\}$ of parts being monitored with a CBM system
${\mathcal {P}}$	Set $\{0,1,\ldots K\}$ of ports
${\mathcal {P}}'$	Index set of trip segments where ${\mathcal {P}}'={\mathcal {P}}\backslash \{0\}$
$s_i$	Service time at port $i\in {\mathcal {P}}$
$\tau _{k}$	Transit time of leg $(k-1,k)$ with standard speed where $k\in {\mathcal {P}}'$
$t_i$	Scheduled arrival time at port $i\in {\mathcal {P}}$
	(Note that $$t_i=t_{i-1}+\tau _{i-1, i}+s_{i-1}$$)
$l_{ji}$	Lead time to deliver part $j\in {\mathcal {N}}$ to port $i\in {\mathcal {P}}$
$c_{ji}$	Procurement cost of part $j\in {\mathcal {N}}$ at port $i \in {\mathcal {P}}$
$f_i$	Fixed maintenance cost at port $i \in {\mathcal {P}}$
$r_j$	Remaining life of the part $j\in {\mathcal {N}}$ from the time at which a warning is received
$w_i$	Delay cost per time unit at port $i\in {\mathcal {P}}$
$p_i$	Penalty cost rate for the extra time spent for the spare part at port $i\in {\mathcal {P}}$
$\xi$	Fraction of the distance from the first of a leg to the point at which a warning is received in that leg for one ore more part(s) ($0<\xi < 1$)
$\alpha$	The ratio of the reduced ship speed over the normal shipping speed ($\alpha <1$)

We model the time of warning received on a leg $k \in {\mathcal {P}}'$ of the journey as a fraction $\xi$ of its length (i.e., distance), for each part $j\in {\mathcal {N}}$. The remaining-life of part $j \in {\mathcal {N}}$ is the distance between the point of warning and the point at which the part is predicted to fail, which corresponds to the time $r_j$ that can be calculated assuming normal (constant) speed of the vessel. In case of a failure, the speed is reduced by a factor of $\alpha < 1$ for the reasons explained earlier. Using these parameters, the increase in transit time on a leg in case of a failure can be calculated as shown in Remark 1 below.

#### Remark 1

If the engine part fails at $\xi$ fraction of leg $(k-1,k)$, then the transit time of the vessel on that leg will be prolonged by $\delta$ time units, where

1 $$\begin{aligned} \delta =\left( \frac{1-\alpha }{\alpha }\right) (1-\xi ) \tau _k. \end{aligned}$$

One can easily verify Eq. (1) by employing simple rules concerning the relationships between time, distance and speed. As an example, consider a leg with standard transit time of $\tau =100$ units and with a zero remaining-life. Suppose that a warning is received and the part fails when the vessel has traversed 60% of the length of the leg ($\xi _1 =0.6$), at which point the speed is reduced to 80% of normal speed ($\alpha =0.8$). The total delay in such a circumstance is $\delta =\frac{1-0.8}{0.8}(1-0.6)100=10$ time units, given that the remaining part of the journey after the failure point is traveled in $\frac{40}{0.8}=50$ instead of 40 time units.

A warning may arise at point $\xi$ of leg $\ell \in {\mathcal {L}}$ during the voyage relating to one or more parts, leading to a decision problem regarding the choice of the port at which spare part should be delivered. Subsequent warnings may also come from the other parts, either on the same leg or subsequent legs, for which the corresponding decision process is repeated with respect to previously made decisions. We denote each optimization problem by PM$(k,\xi )$ which corresponds to a warning received at fraction $\xi$ of the distance traveled at leg $(k-1,k)$. Hence, in our model the optimization problem will be solved in a rolling framework for as many times as the warnings received.

Let $W(k,\xi )$ be the set of parts for which a new warning at $\xi$ fraction of leg $(k-1,k)$ is received. Let $A(k,\xi )$ be the set of parts for which warnings have already been received in previous legs or in leg $(k-1,k)$ before the point $\xi$, and been scheduled to ports to be delivered after leg $(k-1,k)$. We define a binary decision variable $Y_{ji}$ that equals 1 if an order for part $j\in {\mathcal {N}}$ is placed for it to be delivered to port $i\in {\mathcal {P}}$, and 0, otherwise. Let $B_i$ be an intermediate decision variable denoting any extra time that the vessel waits for the arrival of the spare part to a port $i\in {\mathcal {P}}$, and let $D_i$ be any delay accumulated by the vessel, in comparison to the original schedule, by the end of leg $(i-1,i)$ , $i\in {\mathcal {P}}'$. We drop the superscripts $(k,\xi )$ in writing the decision variables for the the sake of notational simplicity.

Vectors $\vec {o}=(o_1,\ldots ,o_N)$, $\vec {\xi }=(\xi _1,\ldots ,\xi _N)$ and $\vec {d}=(d_1,\ldots ,d_N)$ correspond to status of open orders. They respectively retain the legs and fractions at which orders are placed, and subsequently delivered. In particular, $o_j$ denotes the leg number on which the latest order has been placed for part *j* while $d_j$ denotes the port to which the order of part *j* will be delivered. They are updated continuously at each warning point and leg. A tabulated summary of the additional notation is given in Table 2.

**Table 2:** Auxiliary parameters for mathematical modeling

Symbol	Definition
$\phi ^{(k,\xi )}_i$	Total remaining standard transit time from the warning point $\xi$ of leg $(k-1,k)$, $k\in {\mathcal {P}}'$, to port $i\in {\mathcal {P}}, i\ge k$
$\bar{\tau }^{(k,\xi )}_{ji}$	Part of standard transit time of leg $(i-1,i)$ which will be traveled under failure of part $j\in {\mathcal {N}}$ due to the warning received at point $\xi$ of leg $(k-1,k)$ if the part is not replaced until port *i*
$v^{(k,\xi )}_{ji}$	Salvage value of part $j\in {\mathcal {N}}$ whose warning is received at point $\xi$ of leg $(k-1,k)$ in port *i*
$W(k,\xi )$	The set of parts whose warnings are received at the current point on voyage ($\xi$ portion of leg $(k-1,k)$)
$A(k,\xi )$	The set of parts whose warnings are already received before the current point on voyage ($\xi$ portion of leg $(k-1,k)$) and their delivery ports are from/after port *k*
$\vec {o}$	Vector of leg numbers that orders have triggered
$\vec {d}$	Vector of ports to which deliveries are scheduled
$\tilde{B}$	Vector of calculated down-times
$\tilde{D}$	Vector of calculated delays
	* At each phase, *k*, only elements $((\tilde{B}_k,\tilde{D}_k)\ldots (\tilde{B}_K,\tilde{D}_K))$ are updated and the indices prior to *k* remain unchanged

These parameters are mathematically defined below,

2 $$\begin{aligned} \phi ^{(k,\xi )}_{i}=\left\{ \begin{array}{l@{\quad }l} (1-{\xi })\tau _k+\sum \limits _{s=k+1}^{i}\tau _s &{} \text {if } i\ge k\\ 0&{} \text {otherwise}, \end{array} \right. \end{aligned}$$

and

3 $$\begin{aligned} \tau ^{(k,\xi )}_{ji}=\left\{ \begin{array}{l@{\quad }l} \tau _i &{} \text {if } \phi ^{(k,\xi )}_{i-1}\ge r_j\\ \phi ^{(k,\xi )}_i-r_j&{} \text {if } \phi ^{(k,\xi )}_{i-1}\le r_j<\phi ^{(k,\xi )}_{i}\\ 0&{} \text {if } r_j\ge \phi ^{(k,\xi )}_{i}. \end{array} \right. \end{aligned}$$

Calculations in Eq. (3) are straightforward: In the first case, if the remaining-life of part *j* at the point of warning is not greater than the total distance from its warning point to port $(i-1)$, then it will fail before leg $(i-1,i)$ and the entire distance $\tau _i$ of this leg will be traveled at reduced speed. As for the last case, if the remaining-life of part *j* at the point of warning is greater than the remaining distance to port *i*, then the vessel can travel to *i* without failure. In the intermediate cases, the difference between the total distance from the warning point to port *i* and the remaining-life of the part, gives us the part of standard transit time of leg $(i-1,i)$ which will be traveled under the failure of part *j*.

We model the salvage value of a part *j* as a function of its remaining-life at each port. For purposes of mathematical modeling, we normalize the salvage values to be between (0, 1) as below,

4 $$\begin{aligned} v^{(k,\xi )}_{ji}=\left\{ \begin{array}{l@{\quad }l} \frac{\left( r_j-\phi ^{(o_j,\xi _j)}_{i}\right) }{r_j} &{} \text {if } \phi ^{(o_j,\xi _j)}_{i}< r_j\\ 0&{} \text {otherwise}. \end{array} \right. \end{aligned}$$

The salvage value is input to the model in order to schedule delivery of the part as late as possible, particularly if there are multiple optimal solutions. The normalized setting we have used in (4) is particularly useful for when there is lack of real data on salvage values.

### Mathematical programming formulation

Using the notation defined in the previous section, we provide below a mixed integer formulation of the optimisation problem PM($k,\xi$) defined above.

5 $$\begin{aligned} \text {PM(}k,\xi \text {) :}\quad&\text {Minimize } \sum _{i=k}^{K}\left[ f_{i} X_i+\left( \sum \limits _{j\in W(k,\xi )} (c_{ji}+v^{(k,\xi )}_{ji}) Y_{ji}\right) + w_i D_i+p_{i} B_i \right] \end{aligned}$$

subject to

6 $$\begin{aligned} \sum _{i=k}^{K}Y_{ji}&=1&\qquad \forall j\in W(k,\xi ) \end{aligned}$$

7 $$\begin{aligned} X_i&\ge Y_{ji}&\forall j\in W(k,\xi ),\forall i\ge k \end{aligned}$$

8 $$\begin{aligned} X_i&= 1&\text {if }\exists j\in A(k,\xi ) | d_j=i \end{aligned}$$

9 $$\begin{aligned} \delta _{ji}&= \bar{\tau }_{ji}\left( \frac{1-\alpha }{\alpha }\right)&\qquad \forall j\in A(k,\xi ) | i\le d_j \end{aligned}$$

10 $$\begin{aligned} \delta _{ji}&= 0&\forall j\in A(k,\xi ) | i>d_j \end{aligned}$$

11 $$\begin{aligned} \delta _{ji}&= \bar{\tau }_{ji}\left( \frac{1-\alpha }{\alpha }\right) \left( 1-\sum _{t=k}^{i-1}Y_{jt}\right)&\qquad \forall j\in W(k,\xi ) \end{aligned}$$

12 $$\begin{aligned} D_{k-1}&=\tilde{D}_{k-1},&\end{aligned}$$

13 $$\begin{aligned} B_{k-1}&=\tilde{B}_{k-1},&\end{aligned}$$

14 $$\begin{aligned} B_i&=\max \left\{ \max \limits _{j\in A(k,\xi )|d_j=i}\left\{ \left[ \Gamma ^A_{ji}\right] ^+\right\} ,\max \limits _{j\in W(k,\xi )}\left\{ \left[ \Gamma ^W_{ji}\right] ^+\right\} \right\}&\qquad \forall i\in {\mathcal {P}},i\ge k \end{aligned}$$

15 $$\begin{aligned} D_i&= D_{i-1}+B_{i-1}+\max \limits _{j\in W(k,\xi )\cup A(k,\xi )}\delta _{ji}&\forall i\in {\mathcal {P}}, i\ge k \end{aligned}$$

16 $$\begin{aligned} D_{i}, B_i&\ge 0&\forall i\in {\mathcal {P}}, i\ge k \end{aligned}$$

17 $$\begin{aligned} Y_{ji}&\in \{0,1\}&\forall i\in {\mathcal {P}}, i\ge k, j \in W(k,\xi ).&\end{aligned}$$

In model PM($k,\xi$), the decision variables $Y_{ji}$ are defined only for the parts for which a warning has been received at the current point in time, namely parts in the set $W(k,\xi )$. Parts in set $A(k,\xi )$ (open order) or in ${\mathcal {N}}\backslash \left( A(k,\xi )\cup W(k,\xi )\right)$ (healthy parts) are not considered in the above formulation.

The objective function (5) consists of three cost components including those of delay, fixed maintenance cost and augmented procurement cost considering salvage value of the replaced parts. It also incorporates any extra waiting times at pick-up ports as a result of the decisions made at fraction $\xi$ of leg $(k-1,k)$ for spare parts together with the state of the system resulting from the earlier decisions. Constraints (6) ensure placing a new order and scheduling a maintenance for any warning received. Constraints (7) and (8) enforce the model to combine orders by calculating a fixed maintenance cost for the selected ports either from the new warning or from the previous decisions. Equations (9)–(11) calculate the total delay resulting from speed reduction over leg $(i-1,i)$ due to failure of part *j*, assuming all other parts function as normal. At each leg that the problem is executed, the total delay and down-time vectors are retained as vector variable $\tilde{D}$ and $\tilde{B}$. Constraints (12)–(13) update the value of cumulative delay and the down-time prior to the current leg, *k*. Constraint (14) uses two new auxilary variables $\Gamma ^{A}_{ji}$ and $\Gamma ^{W}_{ji}$ for time calculations defined as follows:

18 $$\begin{aligned} \Gamma ^A_{ji}&:= l_{ji}-\phi ^{(o_j,\xi _j)}_i \end{aligned}$$

19 $$\begin{aligned}&\qquad -\sum _{t=o_j}^{i}s_t \end{aligned}$$

20 $$\begin{aligned}&\qquad -\min \left( \max \limits _{j\in {\mathcal {N}}}\bar{\tau }_{j,o_j},\phi ^{(o_j,\xi _j)}_{o_j}\right) \left( \frac{1-\alpha }{\alpha }\right) \end{aligned}$$

21 $$\begin{aligned}&\qquad -\sum _{t=o_j+1}^{k-1}\max \limits _{j\in {\mathcal {N}}}\bar{\tau }_{jt}\left( \frac{1-\alpha }{\alpha }\right) \end{aligned}$$

22 $$\begin{aligned}&\qquad -\sum _{t=k}^{i}\max \limits _{j\in W(k,\xi )\cup A(k,\xi )}\delta _{jt} \end{aligned}$$

23 $$\begin{aligned}&\qquad -\sum _{t=o_j}^{k-1}\tilde{B}_t-\sum _{t=k}^{i-1}B_t. \end{aligned}$$

24 $$\begin{aligned} \Gamma ^W_{ji}&:= l_{ji}Y_{ji}-\phi ^{(k,\xi )}_i \end{aligned}$$

25 $$\begin{aligned}&\qquad -\sum _{t=k}^{i}s_t \end{aligned}$$

26 $$\begin{aligned}&\qquad -\min \left( \max \limits _{j\in {\mathcal {N}}}\bar{\tau }_{j,k},\phi ^{(k,\xi _j)}_{k}\right) \left( \frac{1-\alpha }{\alpha }\right) \end{aligned}$$

27 $$\begin{aligned}&\qquad -\sum _{t=k+1}^{i}\max \limits _{j\in W(k,\xi )\cup A(k,\xi )}\delta _{jt} \end{aligned}$$

28 $$\begin{aligned}&\qquad -\sum _{t=k}^{i-1}B_t. \end{aligned}$$

In particular, $\Gamma ^{A}_{ji}$ is the amount of extra waiting at port *i* for delivery of part *j* which has been ordered at phase $o_j$, is calculated as the difference between its lead-time to port *i*, and the elapsed time from the warning point to that port which includes standard transit time from the warning point to port *i*, $\phi ^{(o_j,\xi )}_i$ in (18), the total service times on these legs in (19), additional transit time due to the speed reduction at the warning leg shown in (20), and subsequent legs prior to the current leg in (21), and in the legs afterwards until *i*, in (22); and finally the down-times within these legs before port *i*, (23). The down-time $\Gamma ^{W}_{ji}$ arising from the set of parts in the new warning set at point $(k,\xi )$ in the current leg is calculated in a similar way except that the terms related to down-time and delays prior to leg $(k-1,k)$ is dropped [see (24)–(27)].

Constraint (14) calculates the additional waiting time for spare parts at port $i \in {\mathcal {P}}'$ as the maximum possible down-time arising either from previously placed orders ($\Gamma ^A_{ji}$), or from any order placed in the current leg ($\Gamma ^W_{ji}$). The reason for the use of the operator $\max$ over all parts in this constraint is to ensure that the time calculation takes into account the spare part last received at port $i \in {\mathcal {P}}'$. Equation (15) calculates the total delay due to speed reduction or any additional waiting for spare parts from the beginning of the journey to leg $(i-1,i)$ at port *i*. Constraints (16) and (17) define the variable domains.

Model PM($k,\xi$) presented above is in the form of a nonlinear mixed integer programming formulation, where the nonlinearities are due to Eqs. (15) and (14). In what follows, we present a linearization that allows the formulation to be solved using a commercial integer linear programming solver.

We first linearize equation (15) using the following set of constraints,

29 $$\begin{aligned} D_i&= D_{i-1}+B_{i-1}+\eta _i&\qquad \forall i\in {\mathcal {P}}, i\ge k \end{aligned}$$

30 $$\begin{aligned} \eta _i&\ge \delta _{ji}&\qquad \forall j\in A(k,\xi )\cup W(k,\xi ), i\in {\mathcal {P}}, i\ge k \end{aligned}$$

31 $$\begin{aligned} \eta _i&\le {\delta }_{ji}+ (1-z_j^i)M&\qquad \forall j\in A(k,\xi )\cup W(k,\xi ), i\in {\mathcal {P}}, i\ge k \end{aligned}$$

32 $$\begin{aligned} \sum \limits _{j\in A(k,\xi )\cup W(k,\xi )}z_j^i&=1&\qquad \forall i\in {\mathcal {P}},i\ge k \end{aligned}$$

33 $$\begin{aligned} z_j^i&\in \{0,1\}&\qquad \forall j\in {\mathcal {N}}, i\in {\mathcal {P}}, \end{aligned}$$

where *M* is a sufficiently large number. In constraint (29), $\eta _i$ is used in place of the expression within the $\max$ operator in (15). Therefore, it must be greater than all the individual terms for which their maximum is sought, which is modeled by constraint (30). Contraints (31)–(33) ensure that only one of the inequalities in (31) is active with the use of the binary $z_j^i$ variable defined for each $j \in A(k,\xi )\cup W(k,\xi )$ and $i\in \{k,\ldots ,K\}$. Together with (30), they ensure that $\eta _i$ will be equal to only one of the values in the argument of $\max$ operator in (15). Using similar arguments, the expression (14) can be linearized using the following set of constraints:

34 $$\begin{aligned} B_i&\ge \Gamma ^A_{ji}&\qquad \forall j\in A(k,\xi ), i\in {\mathcal {P}}, i\ge k \end{aligned}$$

35 $$\begin{aligned} B_i&\ge \Gamma ^W_{ji}&\qquad \forall j\in W(k,\xi ), i\in {\mathcal {P}}, i\ge k \end{aligned}$$

36 $$\begin{aligned} B_i&\le \Gamma ^A_{ji}+(1-u_j^i)M&\qquad \forall j\in A(k,\xi ), i\in {\mathcal {P}}, i\ge k \end{aligned}$$

37 $$\begin{aligned} B_i&\le \Gamma ^W_{ji}+(1-u_j^i)M&\qquad \forall j\in W(k,\xi ), i\in {\mathcal {P}}, i\ge k \end{aligned}$$

38 $$\begin{aligned} B_i&\le Mu^i_0&\qquad \forall i\in {\mathcal {P}}, i\ge k \end{aligned}$$

39 $$\begin{aligned} u^i_0+\sum \limits _{j\in A(k,\xi )\cup W(k,\xi )}u_j^i&=1&\qquad \forall i\in {\mathcal {P}}, i\ge k \end{aligned}$$

40 $$\begin{aligned} u^i_0,u_j^i&\in \{0,1\}&\qquad \forall j\in {\mathcal {N}},i\in {\mathcal {P}}, i\ge k . \end{aligned}$$

The one difference with the linearization of constraints (14) is constraint (38), used to forbid infeasible solutions which will occur if the values of $\Gamma _{ji}^A$ or $\Gamma _{ji}^W$ are negative for all *j*. In such cases, (38) and (39) will set $B_i$ equal to zero.

In the next section, we look at a special case of the problem that results in a simpler and a more efficient solution method.

### Single part management and maintenance scheduling

**Fig. 2:**
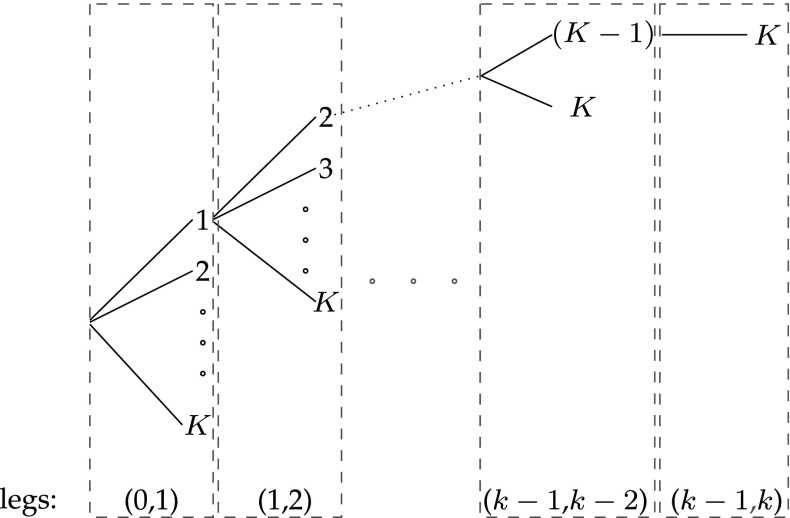
Illustration of the binary tree approach of combining the variables

In this section, we consider a special case of the problem with a single engine part ($N=1$) within the rolling framework described in the previous section. In this case, the problem is triggered when a warning is received from the CBM system on leg $(k-1,k)$, in which case a spare part order is placed to be picked-up at one of the ports $i\in \{k,k+1,\ldots ,K\}$, and no new order is placed until the outstanding order is picked-up. In other words, at most one open order may exist at each time. Provided that a warning is received from the CBM system during the first leg, then *K* different decisions can be made during this phase. Similarly, $K-1$ different decisions can be made during leg (1, 2) provided that the decision in leg (0, 1) is port 1 and a new warning is received during the second leg; otherwise there will be an open order in the second leg and no decision to make. A hierarchy of the tree of decisions that can be made are depicted in Fig. 2.

Let $g^{k}_{i}$ denote the total cost of delay, spare part waiting, procurement and maintenance which arises when warning is received at $\xi ^k$ portion of leg $(k-1,k)$, and where the decision is that the spare part is scheduled to be picked up at port *i*, where $i\in {\mathcal {P}}'$ and $i\ge k$. It is possible to compute the $g^{k}_{i}$ values in a post processing stage as follows,

41 $$\begin{aligned} g^k_{i}=\left\{ \begin{array}{l@{\quad }l} 0,&{} \text {if no warning and } i=k\\ \infty ,&{} \text {if no warning and } i\ne k\\ f_i+c_{1i}+p_i {B^k}_i+\sum _{t=k}^{K}w_i D^k_i,&{} \text {if }\exists \text { a warning at }\xi \in (0,1), i>k,\\ \end{array} \right. \end{aligned}$$

where

42 $$\begin{aligned} D^k_i=\left[ (1-\xi ^k)\tau _k+\sum \limits _{t=k+1}^{i}\tau _t-r_1\right] ^+\left( \frac{1-\alpha }{\alpha }\right) , \qquad k,i\in {\mathcal {P}}', i\ge k, \end{aligned}$$

and

43 $$\begin{aligned} B^k_i=\left[ l_{1i}-\Big ((1-\xi ^k)\tau _k+\sum _{t=k+1}^{i}\tau _t \Big )-D_i^k-\sum _{t=k}^{i}s_t\right] ^+, \qquad k,i\in {\mathcal {P}}', i\ge k. \end{aligned}$$

The following is an illustrative example presenting an application of the concepts developed above.

#### Example 1

**Fig. 3:**
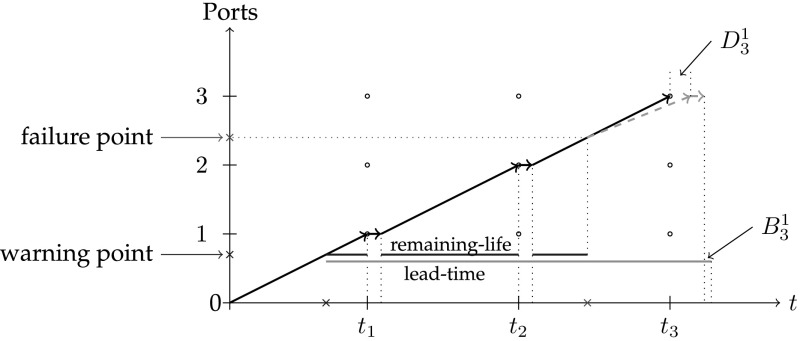
The time-space diagram of the example: at the first leg spare part order is placed for port 3

Consider a route as shown in Fig. 3. Suppose that a warning is received for a single part (shown by the index 1) during leg (0, 1) at point $\xi ^1=0.7$ of the route. Suppose that the transit time is equal to 100 time units for all legs (i.e., $\tau _i=100, i=1,\ldots ,4$), the remaining-life of the part (*r*) is 170 time units and the lead-time of the part procurement for port 3 is 280 time units. All the parameters are given in Table 3.

**Table 3:** Parameters for the illustrative example

*k*	1	2	3
$f_k$	0	0	0
$c_{1k}$	100	110	90
$p_k$	40	45	38
$w_k$	10	15	12
$s_k$	10	10	10
$\tau _k$	100	100	100
$l_{1k}$	200	130	280
$\xi ^k$	0.7	0.9	0.8

We show the steps for calculation of $g^1_{3}$.

$$\begin{aligned} g^1_{3}&=f_3+c_{13}+p_3B_3^1+\big (w_1 D^1_1+ w_2D^1_2+w_3 D^1_3\big )\\&=0+90+38B_3^1+\big (10D^1_1+15D^1_2+12D^1_3\big )\\&=0+90+38\times 5+(10\times 0+ 15\times 0+ 12\times 15)\\&=460, \end{aligned}$$

where

$$\begin{aligned}\begin{array}{l@{\quad }l} D^1_1=0\\ D^1_2=0\\ D^1_3=15=(30+100+100-170)\Big (\frac{1-\,0.8}{0.8}\Big ),\\ \end{array} \end{aligned}$$

and

$$\begin{aligned} B_3^1&=\left[ l_{13}-((1-\xi ^1)\tau _1+\tau _2+\tau _3)-D^1_3-(s_1+s_2+s_3)\right] ^+\\&=\left[ 280-(230)-(15)-(30)\right] ^+\\&=5. \end{aligned}$$

#### Shortest path formulation

**Fig. 4:**
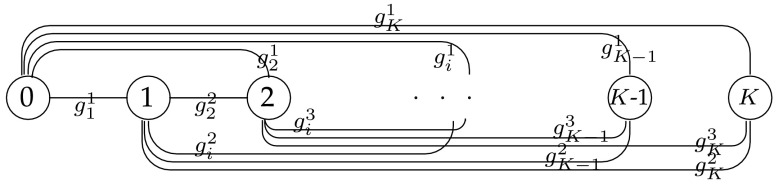
Shortest path network representation

The optimization problem for a single-part can be solved on a graph with $K+1$ nodes, where the distances between any pair of nodes on the graph are given by the $g_i^k$ values that represent the cost of picking up a part at port *i* given a warning is received at phase *k* at point $\xi ^k$. The network is given in Fig. 4. The distance from node *k* and *i* is given by $g^{k+1}_{i}$. The shortest path from node 0 to node *K* in this network is equal to the minimum cost for the problem.

Let $G_i$ be the length of the shortest path from node 0 to node *i*. Then,

44 $$\begin{aligned}&G_0\equiv 0,\end{aligned}$$

45 $$\begin{aligned}&G_i=\min \limits _{\{0\le k<i\}} \{G_k+g^{k+1}_{i}\},\qquad i\in {\mathcal {P}}. \end{aligned}$$

##### Remark 2

In the rolling framework at phase $k=\bar{k}$, only $\xi ^{\bar{k}}$ is observed at node $\bar{k}-1$ of the network, and all $g^{\bar{k}}_i$ are calculated and known while all other $g^{k}_i$ are calculated assuming there is no warning $\forall k>\bar{k}$.

We now present a number of definitions to show the existence of a special policy that can be used for the single part.

##### Definition 1

A function $\phi$ is discretely convex iff $\beta \phi (x)+(1-\beta )\phi (y)\ge \min \limits _{w\in N(z)} \phi (w)$ for $0<\beta <1$ where $z=\beta x+(1-\beta )y$ and $N(x)=\{w\in D: ||w-x||<1 \}$ (Yüceer 2002).

The following properties hold if the fixed ordering and maintenance cost $c_i$ and the lead-times $l_{1i}$ for all $i\in {\mathcal {P}}'$ are stationary or non-increasing over the ports.

##### Remark 3

A non-decreasing (non-increasing) piecewise function over a discrete set is discretely convex.

##### Remark 4

A non-negative linear combination of discretely convex functions is convex.

##### Lemma 1

$g_i^k$ is a discretely convex function over *i*.

##### Proof

$D_i^k$ is non-decreasing and $B_i^k$ is non-increasing in *i* for a stationary lead-time, $l_{1i}=L$, and therefore, they are discretely convex due to Remark 3. Hence, $g_i^k$ is convex due to Remark 4 and the non-negativity of $w_i$ and $p_i$, $\forall i\in {\mathcal {P}}'$. $\square$

##### Proposition 1

There is an optimal $i^*$ which minimizes $g^k_i$ such that $g_{i^*+1}^k\ge g_{i^*}$ and $g_{i^*-1}^k\ge g_{i^*}$.

##### Proof

Due to the convexity of the function $g_i^k$ in *i*, once it starts to increase, then the optimal port cannot be obtained at larger values of $i\in {\mathcal {P}}'$, $i\ge k$. Moreover, since $B_i^k$ is non-increasing in $i\in {\mathcal {P}}'$ and it is constantly zero after a certain $i^+$, where $i^+\le \max \left\{ i, i\ge k:\quad l_{1i}> \Big ((1-\xi ^k)\tau _k+\sum _{t=k+1}^{i}\tau _t \Big )-\sum _{t=k}^{i}s_t\right\}$ using (43). It then suffices to confine the search region of finding optimal spare part pick-up point to $i<i^+$. Thus, arcs $(k-i)$ for $\forall i>i^+$ are pruned, that is to say a myopic approach will identify the optimal port. $\square$

##### Corollary 1

There exists a myopic maintenance policy.

The implication of Corollary 1 is that the optimal port for spare part pick-up is among a limited number of ports ahead of the warning point on the route.

We now discuss further special cases of the single-part problem and discuss the required modifications to the formulation so that it remains valid for these cases. For each of the cases, the costs $g^k_{i}$ would need to be modified in the shortest path formulation.

- If reducing speed from the beginning of a leg is not allowed, it suffices to set $w_i$ for all $i\in {\mathcal {P}}'$ equal to a large number, which will make the appearance of such decisions highly unlikely in an optimal solution due to the minimization operator used in the recursion (45).
- If waiting time for spare part (down-time) is not allowed then such solutions can be penalized by setting the value of $p_k$ to $\infty$.
- If the lead-time $l_{1i}$ and the remaining-life $r_1$ are stochastic, then the network representation of the problem will remain as the shortest path formulation but where the arc costs are stochastic.

## Numerical experiments

In this section we provide a numerical analysis based on simulated data for both the single and multiple part problem. The main objective of the experiments is to quantify the benefits of using a CBM system on costs, indicative of the value of having advance information. Tests are conducted under different scenarios as determined by varying values of the system parameters, which are explained below.

All tests are conducted on an instance of a vessel route with 30 legs, i.e., ${\mathcal {P}}=\{0,\ldots ,30\}$. The additional parameters are set as follows. For each port $i\in {\mathcal {P}}$, the unit delay cost is set equal to $w_i=1$ and the unit penalty cost of waiting for a spare part is set randomly from the interval $p_i\in [10,30]$. Transit times are drawn from a uniform distribution in the interval [80, 200], and the service time at each port $i\in {\mathcal {P}}'$ is randomly set from the interval $s_i\in [15,25]$, which translates into a scheduled arrival time at each port as explained earlier. The failure warning points $\xi$ are generated from a uniform distribution in (0, 1) for each leg with 50% probability for parts in healthy status. We have generated 10 realizations for warning points and present the results averaged over 10 runs. In the absence of real data, the lead-time and remaining-life time values are generated as a function of the average transit time as one of $\{0,\bar{\tau },2\bar{\tau },3\bar{\tau }\}$, so that the units are compatible. The rate of speed reduction is selected from the set $\alpha =\{0.3,0.4,0.5,0.6,0.7,0.8\}$. The fixed unit maintenance/order cost $f_i$ is randomly chosen from the uniform interval [35, 65] while the procurement cost $c_{ji}$ of each part is randomly selected from the interval [5, 10] for each $j\in {\mathcal {N}}$ and $i\in {\mathcal {P}}'$.

**Fig. 5:**
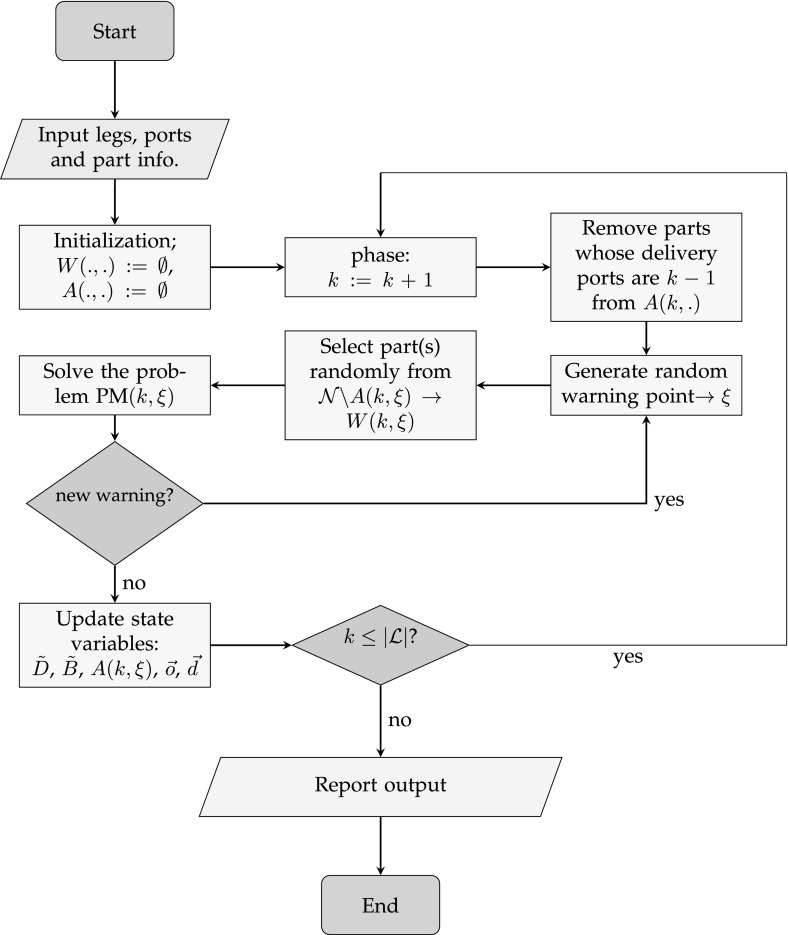
The flowchart of the simulation approach

The mathematical programming model is embedded in a Monte-Carlo simulation of the engine parts malfunctions. Each engine part may give at most one warning (if in healthy status) in a leg. In the extreme case, if all of the parts give warnings at each leg and are fixed in the nearest port, then at most $|{\mathcal {L}}|\times |{\mathcal {N}}|$ optimization models will be solved within the entire simulation. All maintenance and replacement activities are performed only at ports, hence we define each leg of the route as a problem phase. At the end of each phase, all previously made decisions are fixed, and the problem is re-solved for the subsequent leg on the journey. Figure 5 depicts the abstract flowchart of our model implementation from which our numerical results are obtained.

All the experiments were conducted on a personal computer with 2.30GHz of CPU and 2GB of RAM. The model was coded in C++ using Concert Technology, using CPLEX 12.6 as the solver. As each instance was solved in a matter of a few seconds, we do not report detailed computational times here. We present the results in two main sections, first concerning a single part, and then with multiple parts.

### Performance measures

In order to investigate the behavior of the system for maintenance decisions, the following average performance indicators are used: (i) number of warnings ($\#wn$) and number of ports where maintenance is performed which we refer to as *setups*, (ii) number of legs traveled from the warning point to the delivery port, i.e., order-to-delivery (*od*), (iii) total deviation percentage from the schedule ($dev\%$), (iv) number of down-times ($\#dt$) and (v) its ratio to total scheduled journey time (*rdt*). All values reported below are the averages over the 10 realizations. We discuss the sensitivity of these performance measures to the rate $\alpha$ of reduction of speed, the lead-time *l* and the remaining-life *r*.

### Experiments with a single part

We have generated our problem instances for different values of lead-time (*l*), remaining-life at the warning point (*r*), and reduction in speed ($\alpha$). A total of $l\times r\times 10 \times \alpha$ = 4$\times$4$\times$10$\times$6 = 960 problem instances were simulated and solved for the single part problem. Optimal solutions could be produced by using the DP algorithm described in Sect. 3.3 with ${\mathcal {O}}(K)$ complexity order at each decision point, although for our purposes it sufficed to use CPLEX 12.6. The average results are given summarized in Table 4 which include the five performance measures. In particular, the first two columns show the lead-time and remaining-life values, and the two remaining columns show two different rates of speed reduction. We discuss the findings below:

**Table 4:** Simulation result for different lead-time, remaining-life and $\alpha$ values

	*r*	$\alpha =0.3$					$\alpha =0.4$				
		$\#wn$	*od*	$dev\%$	*rdt*	$\#dt$	$\#wn$	$o-d$	$dev\%$	*rdt*	$\#dt$
$l=0$	0	14	0.478	47.4	0	0	14	0.5	30.4	0	0
	$\bar{\tau }$	13	0.532	0.7	0	0	13	0.5	0.5	0	0
	2$\bar{\tau }$	11	1.048	0	0	0	11	1	0	0	0
	3$\bar{\tau }$	9	1.572	0	0	0	9	1.6	0	0	0
$l=\bar{\tau }$	0	13	0.556	54.7	3	3	13	0.6	38.9	2.9	3
	$\bar{\tau }$	12	0.958	13.2	3.2	4	11	1.1	10.8	1.9	3
	2$\bar{\tau }$	10	1.34	0	0	0	10	1.3	0	0	0
	3$\bar{\tau }$	9	1.978	0	0	0	9	2	0	0	0
$l=2\bar{\tau }$	0	12	0.786	71.4	5.7	3	11	1	54.3	3.4	3
	$\bar{\tau }$	10	1.398	34.9	4.4	3	10	1.6	28.6	1.3	1
	2$\bar{\tau }$	9	1.855	8.9	1.3	2	9	1.9	7.2	0.9	2
	3$\bar{\tau }$	7	2.285	0	0	0	7	2.3	0	0	0
$l=3\bar{\tau }$	0	11	1.09	88.3	4.8	3	10	1.4	63.8	2.4	2
	$\bar{\tau }$	9	1.681	53.8	3.8	3	9	1.9	41	2.1	2
	2$\bar{\tau }$	7	2.337	24.2	2.7	2	7	2.4	16.8	1.7	2
	3$\bar{\tau }$	6	2.765	2.8	0.3	1	6	2.8	1.9	0.3	1
	*r*	$\alpha =0.5$					$\alpha =0.6$				
		$\#wn$	*od*	$dev\%$	*rdt*	$\#dt$	$\#wn$	$o-d$	$dev\%$	*rdt*	$\#dt$
$l=0$	0	14	0.478	20.3	0	0	14	0.5	13.5	0	0
	$\bar{\tau }$	13	0.532	0.3	0	0	13	0.6	0.2	0	0
	2$\bar{\tau }$	11	1.048	0	0	0	11	1	0	0	0
	3$\bar{\tau }$	9	1.572	0	0	0	9	1.6	0	0	0
$l=\bar{\tau }$	0	12	0.775	29.6	1.5	2	12	0.9	21.4	0.7	1
	$\bar{\tau }$	11	1.131	8	0.9	2	10	1.2	6.3	0.4	1
	2$\bar{\tau }$	10	1.357	0	0	0	10	1.4	0	0	0
	3$\bar{\tau }$	9	1.978	0	0	0	9	2	0	0	0
$l=2\bar{\tau }$	0	10	1.225	39.5	1.6	2	10	1.4	29.3	0.5	1
	$\bar{\tau }$	9	1.638	20.8	0.7	1	9	1.8	15.2	0.3	1
	2$\bar{\tau }$	9	1.97	5.4	0.6	1	8	2	3.6	0.3	1
	3$\bar{\tau }$	7	2.285	0	0	0	7	2.3	0	0	0
$l=3\bar{\tau }$	0	9	1.638	48.9	0.7	1	9	2	36	0.7	1
	$\bar{\tau }$	8	2.051	28.7	1.2	2	7	2.3	19.5	0.3	1
	2$\bar{\tau }$	7	2.558	13.4	0.7	1	6	2.6	9.1	0.2	1
	3$\bar{\tau }$	6	2.801	1.5	0.2	1	6	2.8	1.1	0.2	1
	*r*	$\alpha =0.7$					$\alpha =0.8$				
		$\#wn$	*od*	$dev\%$	*rdt*	$\#dt$	$\#wn$	$o-d$	$dev\%$	*rdt*	$\#dt$
$l=0$	0	14	0.478	8.7	0	0	14	0.5	5	0	0
	$\bar{\tau }$	13	0.552	0.1	0	0	13	0.6	0.1	0	0
	2$\bar{\tau }$	11	1.055	0	0	0	11	1.1	0	0	0
	3$\bar{\tau }$	9	1.599	0	0	0	9	1.6	0	0	0
$l=\bar{\tau }$	0	11	0.979	15	0.4	1	11	1.1	9.3	0	0
	$\bar{\tau }$	10	1.236	4.2	0.4	1	10	1.3	2.7	0	0
	2$\bar{\tau }$	10	1.357	0	0	0	10	1.4	0	0	0
	3$\bar{\tau }$	9	1.978	0	0	0	9	2	0	0	0
$l=2\bar{\tau }$	0	10	1.592	20.6	0.2	1	9	1.7	12.3	0	0
	$\bar{\tau }$	9	1.8	10.4	0.4	1	9	2	7	0.1	1
	2$\bar{\tau }$	8	2.01	2.4	0.1	1	8	2.1	1.5	0	0
	3$\bar{\tau }$	7	2.285	0	0	0	7	2.3	0	0	0
$l=3\bar{\tau }$	0	7	2.209	22.4	0.2	1	7	2.4	13.2	0.1	1
	$\bar{\tau }$	7	2.435	13.6	0.2	1	6	2.6	8.3	0	0
	2$\bar{\tau }$	6	2.678	6.2	0	1	6	2.7	3.8	0	0
	3$\bar{\tau }$	6	2.82	0.8	0.2	1	6	2.9	0.7	0	1

**Fig. 6:**
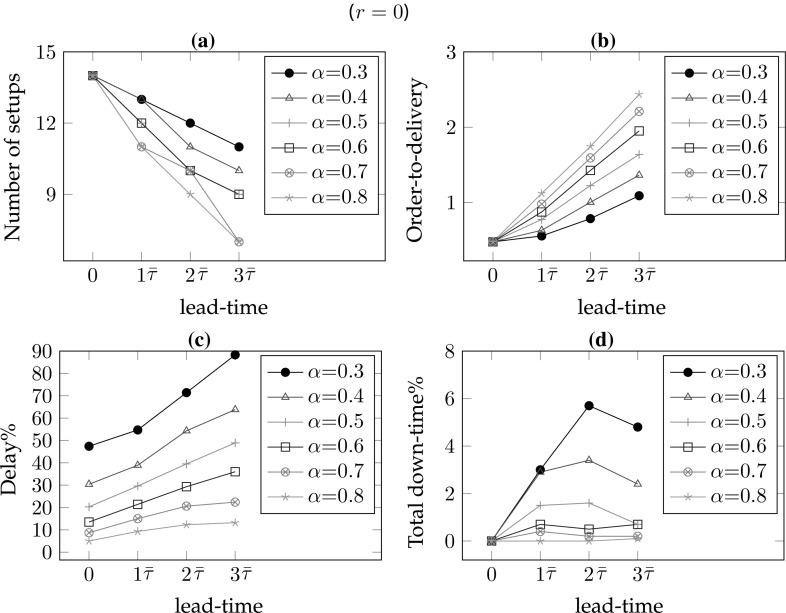
Sensitivity of the performance measures to lead-time and $\alpha$ for $r=0$

**Fig. 7:**
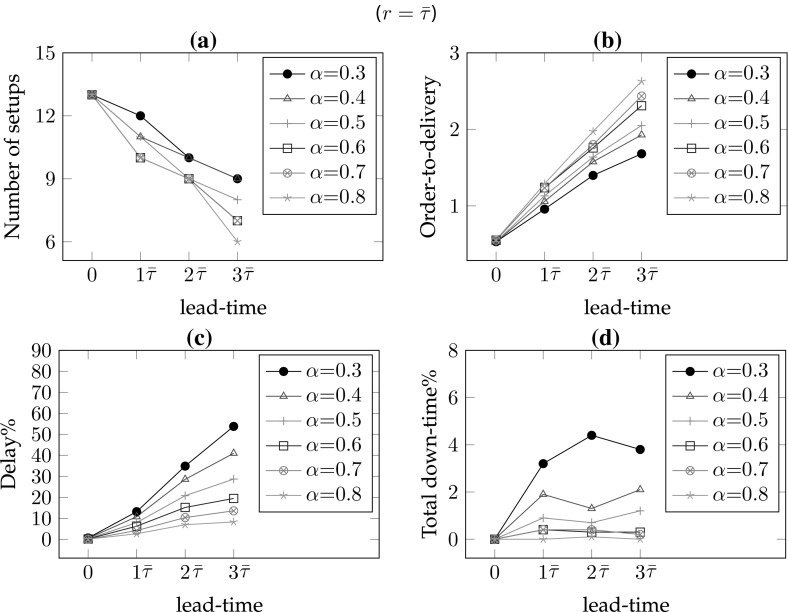
Sensitivity of the performance measures to lead-time and $\alpha$ for $r=\bar{\tau }$

**Fig. 8:**
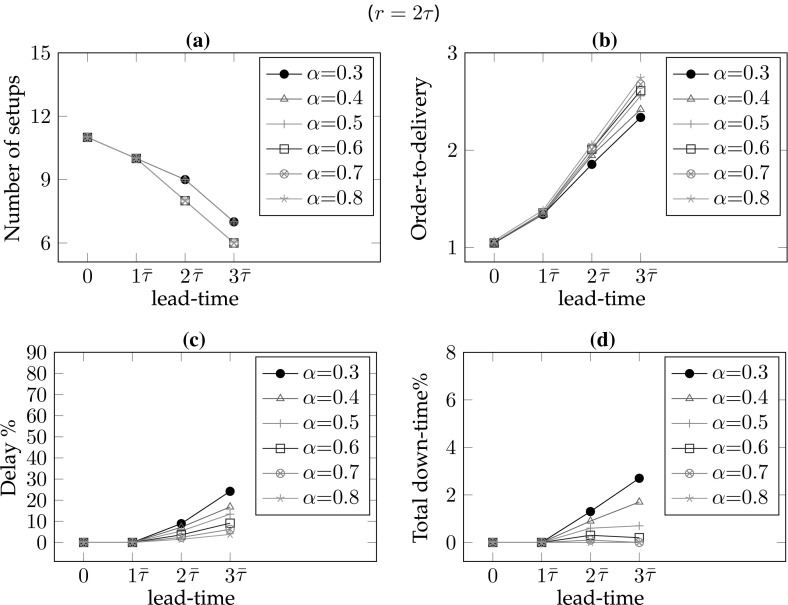
Sensitivity of the performance measures to lead-time and $\alpha$ for $r=2\bar{\tau }$

**Fig. 9:**
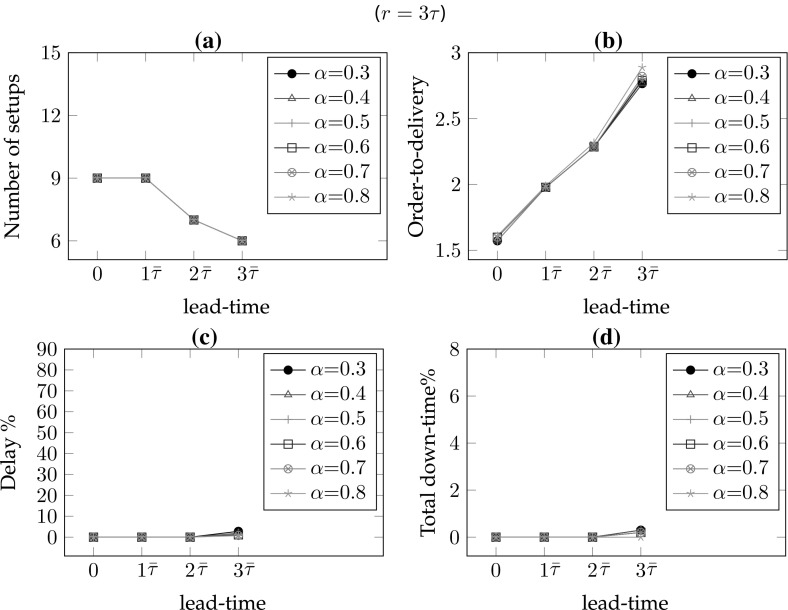
Sensitivity of the performance measures to lead-time and $\alpha$ for $r=3\bar{\tau }$

- *# of warnings/setups* In the single part case, the number of setups equals the number of warnings. As the frequency of maintenance increases, the number of setups also increases. Hence, for lower values of the lead-time and the remaining-life, there are more setups so $\#wn$ is decreasing in *r* for all lead-times (*l*) and decreasing in *l* for all remaining-life (*r*) values. In addition, increasing values of $\alpha$ result in smaller delays and consequently fewer maintenance interventions; hence, $\#wn$ is decreasing in $\alpha$ for all *r* and *l* values (see Figs. 6a, 7a, 8a, 9a).
- *Order-to-delivery* This measure is sensitive mostly to the lead-time with an increasing trend in *l* as seen in Figs. 6b, 7b, 8b, 9b. However, it is decreasing in $\alpha$ because the higher the value of $\alpha$, the smaller the delay cost, for which reason it is preferable to perform maintenance operations at later ports on the route. The results suggest that the order-to-delivery measure is bounded above by the lead-time value. It is also increasing in *r*, and for the larger values of *r* it converges to the lead-time for all values of $\alpha$ (see Fig. 9d).
- *Delay percentage* Larger values of *r* imply less travel made with reduced speed. For larger values of $\alpha$ lower delays are observed due to the reduced speed. It is therefore not surprising that the values in the columns $dev\%$ show a decreasing pattern in *r* and $\alpha$ irrespective of lead-times. On the other hand, larger lead-time values result in increased possibility of down-times which are subsumed in the delays [see Eq. (15)], for which reason $dev\%$ values are increasing in *l* (see also Figs. 6c, 7c, 8c, 9c). The magnitude of delays without a CBM system is significantly higher than the case where there is a CBM system in place. In particular, even under large lead-times ($l=3\bar{\tau }$), the average deviations from the schedule in the absence of a CBM system are equal to 47.4, 30.4, 20.3, 13.5, 8.7 and 5 (for $r=0$), respectively, for $\alpha = 0.3, 0.4, 0.5, 0.6, 0.7 \,and\, 0.8,$ and are largely reduced to 2.8, 1.9, 1.5, 1.1, 0.8 and 0.7 (for $r=3\bar{\tau }$), respectively, when a CBM system is in place. An interesting observation is that when $r>l$ then the delays can be prevented and are insensitive to $\alpha$. For instance all delay deviations in Fig. 6c are distinct for varying $\alpha$ and *l* values, while they converge to zero for $l=0$ and for all $\alpha$, as shown in Fig. 7c because ($r=$)$\bar{\tau }>l(=0)$. Similarly, and as seen in Figs. 8c ($r=2\bar{\tau }$) and 9c ($r=3\bar{\tau }$), the first two and the first three values of *l* result in zero delay for all $\alpha$ values because $r>l$. Therefore, if a failure can be predicted more than *l* time units prior to its failure, it is possible even in theory that any delay can be avoided.
- *Down-times* The order-to-delivery intervals are increasing in *r*, as expected, given that larger values of *r* make it reasonable to schedule the delivery of the spare part to later ports on the route. The average number of down-times, $\#dt$, and the ratio of total down-times to total travel time, *rdt*, tend to have a non-increasing pattern in *r* and *l* although there may be exceptions (e.g., comparing $r=0$ and $r=\bar{\tau }$ for $\alpha =0.5$, $l=3\bar{\tau }$: 0.7 vs. 1.2). These, however, are not unusual results because down-times are included in total delays and when the delays are compared in such cases we find that there is a significant reduction. The results suggest that it might be preferable to have longer down-times as opposed to delays arising from speed reduction, in order to reduce the total delay.

Figures 6, 7, 8 and 9 correspond to the results given in Table 4, where each figure corresponds to a specific value of remaining-life. In each figure, graph (a) shows the average number of setups; graph (b) illustrates the average number of legs traveled in the warned status; graph (c) presents the average total deviation percentage from the schedule, namely delay %, and (d) shows the total down-time as a percentage of total travel time. The horizontal axis in all of the graphs corresponds to the lead-time which is calibrated in terms of average transit times of legs. The trends of all performance measures are plotted for all $\alpha$ values with different colors and marks. The scenario with $r=0$ shown in Fig. 6 corresponds to the situation in which there is no CBM system in place, and where a part fails immediately at the time of the warning. A comparison of the four plots in this figure highlights the role of the CBM system in improving the system performance. For instance, in the absence of a CBM system, the total delay % in the extreme case of $\alpha = 0.3$ can be up to 90% (see Fig. 6c) while the corresponding values for $r=\bar{\tau }$, $r=2\bar{\tau }$ and $r=3\bar{\tau }$ are almost 50, 25 and 5%, respectively (see Figs. 7c, 8c, 9c).

One other interesting point is the insensitivity of all performance measures to $\alpha$ when the remaining-life at the warning point is sufficiently large, as can be seen in Fig. 9. In other words, if there is a very reliable CBM system capable of predicting part break-down sufficiently long in advance,^1^ then delays and down-times can –to a great extent– be prevented. As the discussion above suggests, the delay is more sensitive to $\alpha$ when the lead-time is greater than the remaining-life. When the remaining-life and the lead-time are in the same order of magnitude, the delay is relatively less sensitive to the value of $\alpha$. In those cases, lower values of $\alpha$ lead to more maintenance and procurement interventions needed to prevent significant delay costs.

### Experiments with multiple parts

This section extends the numerical analysis to five parts, for which the remaining-life values at the point of warning are set as ($r_1,\ldots ,r_5$) = $(\bar{\tau },\bar{\tau },2\bar{\tau },3\bar{\tau },4\bar{\tau })$. In order to investigate the effect of remaining-life, we set $r_j=0$ for one value of *j* at a time whilst keeping the other values intact. The same lead-time values are considered for all parts for all the ports, namely $l_{ji}=l$ for $j\in {\mathcal {N}}$, $i\in {\mathcal {P}}'$ where $l=\kappa \bar{\tau }$ where $\kappa \in \{0,1,2,3,4\}$. In this case, two values of $\alpha \in \{0.4, 0.7\}$ are used. The rest of the parameters are as described for the single-part case, resulting in a total of $l\times r\times 10\times \alpha$ = 5$\times$6$\times$10$\times$2 = 600 problem instances altogether, where we perform 10 runs of the simulation for each instance.

In addition to the performance measures described in the previous section, we also introduce two others, namely, the number $\#Lw$ of legs in which any warnings are received, and the number $\#stp$ of setups. In contrast to the single part case, the number of setups is not equal to the number of warnings in the multiple part system. The number of legs traveled in the warned status (order-to-delivery) are specified for each part separately using $od_j$ labels $j\in \{1,\ldots 5\}$. Similarly, number of warnings for each part are presented separately via $\#wn_j$, $j\in \{1,\ldots 5\}$ labels in Tables 5 and 6. Below we discuss each of our performance measures.

**Table 5:** Performance measures average for 10 replication of simulation for a 5-part system with $\alpha =0.4$

$\vec {r}$	$\kappa$	$\#wn$	$\#Lw$	$\#stp$	$od_1$	$od_2$	$od_3$	$od_4$	$od_5$	$\#wn_1$	$\#wn_2$	$\#wn_3$	$\#wn_4$	$\#wn_5$	$dev\%$	*rdt*	$\#dt$
(0, 0, 0, 0, 0)	0	58.1	14.3	28.6	0.5	0.5	0.5	0.5	0.5	11.0	10.9	11.3	12.4	12.4	33.9	0.0	0.0
	1	54.0	14.2	29.3	0.6	0.6	0.7	0.6	0.7	10.4	9.7	10.2	12.0	11.7	52.0	4.8	4.4
	2	43.8	12.2	29.0	0.9	1.0	1.0	1.1	1.1	7.8	8.8	8.8	9.3	9.1	63.8	2.7	3.0
	3	43.1	12.7	29.0	1.4	1.4	1.4	1.4	1.4	7.8	8.3	8.3	9.2	9.4	81.5	2.8	3.0
	4	37.4	10.9	28.9	1.6	1.6	1.6	1.6	1.6	7.0	7.3	7.6	7.6	8.0	87.9	11.0	4.7
$(\bar{\tau },\bar{\tau },2\bar{\tau },3\bar{\tau },0)$	0	52.6	15.2	28.6	0.5	0.5	1.5	2.6	0.5	11.7	11.6	8.7	6.9	13.8	27.7	0.0	0.0
	1	47.0	14.1	28.9	0.7	0.7	1.5	2.5	0.7	9.6	9.9	8.8	6.4	12.3	41.2	3.7	3.9
	2	35.8	11.6	27.9	1.2	1.2	1.5	2.6	1.2	7.0	7.4	6.9	6.0	8.4	46.9	1.3	1.6
	3	41.3	13.2	29.3	1.5	1.4	1.6	2.5	1.4	7.8	8.4	8.3	7.1	9.7	75.9	3.1	2.6
	4	37.8	11.9	29.2	2.0	1.9	2.1	2.6	1.9	7.2	7.6	7.3	7.2	8.4	80.2	3.2	2.7
$(\bar{\tau },\bar{\tau },2\bar{\tau },0,4\bar{\tau })$	0	49.1	14.7	28.6	0.5	0.5	1.5	0.5	3.5	10.8	11.0	8.4	13.4	5.4	27.6	0.0	0.0
	1	45.6	14.0	29.2	0.8	0.7	1.5	0.6	3.5	9.3	9.9	8.2	12.0	6.1	42.5	3.7	3.7
	2	38.1	12.0	28.4	1.2	1.2	1.6	1.2	3.5	7.7	8.1	7.8	9.3	5.2	51.3	1.5	2.0
	3	39.6	12.6	28.0	1.5	1.5	1.7	1.5	3.5	7.8	8.4	8.4	9.1	5.8	67.9	1.5	2.6
	4	34.8	11.6	29.4	1.9	2.0	2.0	1.8	3.5	6.9	7.3	7.0	7.8	5.8	80.1	3.3	2.0
$(\bar{\tau },\bar{\tau },0,3\bar{\tau },4\bar{\tau })$	0	49.8	15.6	28.6	0.5	0.5	0.5	2.5	3.5	11.7	11.8	13.7	6.9	5.8	28.0	0.0	0.0
	1	44.7	14.1	29.0	0.7	0.8	0.7	2.5	3.5	9.9	10.2	11.6	7.4	5.6	40.5	2.2	2.8
	2	39.1	12.8	28.3	1.1	1.2	1.1	2.6	3.5	8.2	9.1	10.0	6.3	5.4	55.0	1.6	2.0
	3	37.6	13.4	29.0	1.5	1.6	1.5	2.5	3.4	7.6	8.1	8.8	7.0	6.1	66.5	2.5	3.2
	4	33.9	12.8	28.9	1.8	1.8	1.8	2.5	3.5	6.4	7.2	8.0	6.8	5.4	78.9	5.4	2.9
$(\bar{\tau },0,2\bar{\tau },3\bar{\tau },4\bar{\tau })$	0	42.0	15.0	28.6	0.5	0.5	1.5	2.5	3.5	10.7	11.2	8.0	6.9	5.2	25.7	0.0	0.0
	1	41.1	14.2	28.2	0.8	0.7	1.5	2.5	3.4	9.4	10.3	8.7	7.0	5.7	38.4	2.1	2.1
	2	37.7	13.1	28.0	1.3	1.1	1.5	2.5	3.5	8.0	8.9	8.3	6.9	5.6	51.9	0.8	1.3
	3	36.9	13.3	29.4	1.5	1.5	1.7	2.5	3.5	7.4	8.4	8.2	7.0	5.8	65.3	2.2	2.7
	4	36.4	13.2	29.1	1.8	1.7	2.0	2.5	3.5	7.4	8.2	7.6	7.3	5.9	77.1	2.4	2.4
$(\bar{\tau },\bar{\tau },2\bar{\tau },3\bar{\tau },4\bar{\tau })$	0	41.8	14.9	28.6	0.5	0.5	1.5	2.5	3.5	10.7	11.0	8.0	6.9	5.2	0.0	0.0	0.0
	1	38.8	13.4	28.0	0.9	1.0	1.5	2.5	3.5	8.7	9.1	8.6	6.8	5.7	8.4	2.2	4.1
	2	35.4	12.0	29.1	1.5	1.5	1.5	2.5	3.5	7.0	7.8	8.3	6.9	5.4	24.4	2.5	2.2
	3	35.7	12.9	28.7	1.8	1.7	1.9	2.5	3.5	7.2	8.1	7.3	7.2	5.8	41.3	6.3	3.1
	4	34.9	12.8	28.3	2.2	2.1	2.4	2.6	3.5	6.9	7.4	7.2	7.1	6.2	54.9	3.2	2.7

**Table 6:** Performance measures average for 10 replication of simulation for a 5-part system with $\alpha =0.7$

$\vec {r}$	$\kappa$	$\#wn$	$\#Lw$	$\#stp$	$od_1$	$od_2$	$od_3$	$od_4$	$od_5$	$\#wn_1$	$\#wn_2$	$\#wn_3$	$\#wn_4$	$\#wn_5$	$dev\%$	*rdt*	$\#dt$
(0, 0, 0, 0, 0)	0	58.1	14.3	28.6	0.5	0.5	0.5	0.5	0.5	11.0	10.9	11.3	12.4	12.4	9.7	0.0	0.0
	1	46.3	13.3	29.3	0.9	1.0	1.0	1.0	1.0	9.0	8.2	8.9	10.1	10.1	19.8	0.5	2.1
	2	39.3	11.9	28.9	1.5	1.5	1.5	1.6	1.6	6.8	7.4	8.4	8.1	8.6	23.8	0.9	3.1
	3	34.8	11.1	29.4	2.2	2.1	2.2	2.1	2.2	6.3	6.7	7.1	7.2	7.4	29.6	0.7	2.0
	4	31.1	11.6	28.8	2.8	2.8	2.9	2.9	3.0	6.0	6.1	6.2	6.3	6.4	35.9	0.9	2.4
$(\bar{\tau },\bar{\tau },2\bar{\tau },3\bar{\tau },0)$	0	52.6	15.2	28.6	0.5	0.5	1.5	2.6	0.5	11.7	11.6	8.7	6.9	13.8	7.9	0.0	0.0
	1	45.4	13.7	28.9	0.9	1.0	1.5	2.5	1.0	9.4	9.6	9.0	6.6	10.9	15.9	0.2	1.6
	2	38.3	13.0	29.3	1.6	1.6	1.6	2.5	1.5	7.0	7.7	8.2	6.6	8.9	20.1	0.7	2.3
	3	33.7	10.9	29.2	2.2	2.3	2.3	2.5	2.2	6.1	6.7	7.2	6.4	7.2	25.3	0.5	1.7
	4	31.7	11.1	28.8	2.9	2.9	3.0	3.1	3.0	5.9	6.4	6.3	6.4	6.6	30.7	0.9	1.3
$(\bar{\tau },\bar{\tau },2\bar{\tau },0,4\bar{\tau })$	0	49.1	14.7	28.6	0.5	0.5	1.5	0.5	3.5	10.8	11.0	8.4	13.4	5.4	7.8	0.0	0.0
	1	46.3	13.8	29.2	1.0	1.0	1.5	1.0	3.5	9.9	10.1	9.2	11.3	5.8	16.8	0.2	1.1
	2	35.3	12.0	28.0	1.7	1.7	1.7	1.7	3.5	6.6	7.1	7.6	8.3	5.8	20.4	0.5	1.8
	3	33.8	11.7	29.2	2.2	2.3	2.3	2.2	3.5	6.3	7.1	7.2	7.3	5.8	27.0	1.3	1.7
	4	28.9	11.6	29.2	2.9	3.0	3.1	3.0	3.5	5.4	6.0	5.6	5.9	6.0	29.5	0.4	1.8
$(\bar{\tau },\bar{\tau },0,3\bar{\tau },4\bar{\tau })$	0	49.8	15.6	28.6	0.5	0.5	0.5	2.5	3.5	11.7	11.8	13.7	6.9	5.8	7.9	0.0	0.0
	1	42.8	14.4	29.0	1.0	1.1	1.0	2.5	3.5	9.4	9.6	10.8	7.2	5.8	16.0	0.5	1.8
	2	34.8	11.4	27.4	1.6	1.7	1.6	2.5	3.5	7.0	7.9	7.9	6.3	5.7	19.8	0.5	1.9
	3	33.1	11.6	28.7	2.3	2.3	2.3	2.6	3.5	6.4	7.0	7.0	6.8	5.9	25.0	1.8	1.6
	4	31.9	12.8	29.2	2.8	3.0	2.9	3.2	3.5	5.9	6.3	6.8	6.6	6.3	33.2	2.2	2.1
$(\bar{\tau },0,2\bar{\tau },3\bar{\tau },4\bar{\tau })$	0	42.0	15.0	28.6	0.5	0.5	1.5	2.5	3.5	10.7	11.2	8.0	6.9	5.2	7.3	0.0	0.0
	1	39.1	13.7	28.2	1.0	1.0	1.5	2.5	3.5	8.8	9.2	8.3	7.0	5.8	12.9	0.4	1.2
	2	34.8	11.8	29.3	1.6	1.6	1.7	2.5	3.5	6.7	7.9	8.0	6.7	5.6	18.4	0.5	2.0
	3	33.9	12.1	28.3	2.3	2.3	2.4	2.5	3.5	6.9	7.0	7.3	6.8	5.9	24.8	0.3	1.3
	4	30.6	11.0	28.7	2.9	2.9	3.1	3.2	3.5	5.9	6.2	6.1	6.2	6.1	29.4	1.2	1.7
$(\bar{\tau },\bar{\tau },2\bar{\tau },3\bar{\tau },4\bar{\tau })$	0	41.8	14.9	28.6	0.5	0.5	1.5	2.5	3.5	10.7	11.0	8.0	6.9	5.2	0.0	0.0	0.0
	1	38.1	13.3	28.0	1.2	1.2	1.5	2.5	3.5	8.3	8.4	8.9	6.9	5.6	4.2	0.2	1.3
	2	34.2	12.0	29.1	1.7	1.8	1.8	2.5	3.4	7.0	7.4	7.6	6.8	5.4	11.4	1.1	2.4
	3	33.3	11.7	29.0	2.4	2.5	2.5	2.5	3.4	6.6	6.7	7.0	7.0	6.1	18.6	1.1	1.9
	4	30.6	10.7	29.0	3.0	3.1	3.2	3.2	3.4	5.9	6.2	6.1	6.1	6.2	22.9	0.5	1.9

**Fig. 10:**
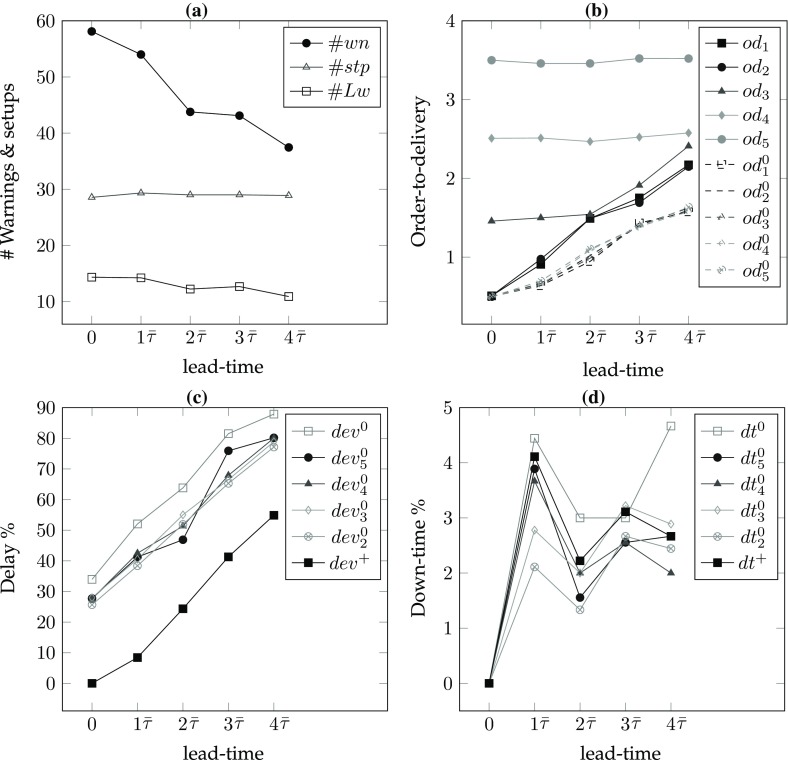
Sensitivity analysis of the performance measures for a 5-part system with 30 legs and $\alpha =0.4$

**Fig. 11:**
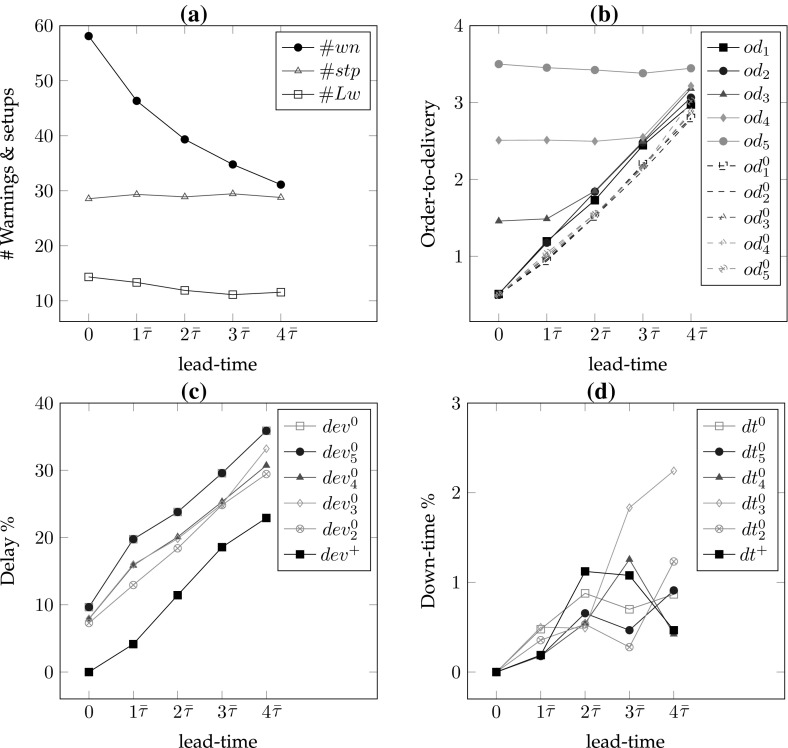
Sensitivity analysis of the performance measures for a 5-part system with 30 legs and $\alpha =0.7$

- *Number of warnings, setups, and legs with warnings* The number of warnings are decreasing in *l* (for all values of remaining-life) and $\alpha$. This is due to the fact that for shorter values of the lead-time the parts are replaced quicker, but might fail again given that the warnings are generated irrespective of remaining-life. Similarly, the number of legs in which new warnings are received is also decreasing in *l* for both $\alpha$ values while $\#stp$ and $\#Lw$ do not follow any increasing or decreasing pattern in *l* or $\alpha$. The trends of these measures are illustrated in Figs. 10a and 11a. Looking at the individual number of warnings for each part separately in columns $\#wn_j$, $j\in \{1,\ldots ,5\}$ we observe that the number of warnings for the parts with larger values of *r* is lower than that of the other parts.
- *Order-to-delivery* Looking at the data in columns $od_1\ldots od_5$ in Tables 5 and 6 we find that the order-to-delivery values of the parts with a small remaining-life are low. In particular the parts with zero remaining-life will be replaced quicker than others. It is worthwhile to mention that, similar to the single part system, the $od_1\ldots od_5$ values are bounded above by the lead-time values. In Figs. 10b and 11b the order-to-delivery values of parts with positive remaining-life are compared to those assuming zero remaining-life, namely when no CBM information is available for that part. For instance the dashed plot with legend $od_1^0$ corresponds to the order-delivery of part 1 if there is no CBM system in place for this part. Parts 5 and 4 (the brown and the green plots) have the greatest remaining-life values (when they are positive), therefore their *od* values are either less sensitive or insensitive to the lead-time.
- *Delay percentage* Similar to the observations in the single part system, we also observe here that the delay percentage is decreasing in *l* and $\alpha$ for each group of the remaining-life values as shown in Tables 5 and 6. An interesting observation here is that the effectiveness of the CBM system for a part with shorter remaining-life may be more than that of a part with longer remaining-life. For instance, the delay % is zero for a part with a zero lead-time when $\alpha =0.4$ and when all the remaining-life values are positive. When $r_2=0$, $r_3=0$, $r_4=0$, $r_5=0$, however, the delays are 25.7, 28, 27.6, 27.7%, respectively. Overall, it is preferable to have advance information for each part as otherwise the delays will increase (see column $dev\%$ in Tables 5 and 6). Figures 10c and 11c depict the comparison of average delay percentages when all the remaining-life values are positive ($dev^+$) for cases where parts $j=5,4,3,2$ lack a CBM system ($dev^0_j$), or when no CBM system exists for any of the parts ($dev^0$).
- *Down-times* Similar to the single part system, the average total down-time does not follow a monotone pattern over the lead-time values or $\alpha$ (see Figs. 10d, 11d). The reasoning for the single part case is also valid here, namely to incur more down times so as to reduce the overall delay.

## Conclusions

In this study we have introduced, formulated and studied a spare part management problem arising in the maritime sector in which the failure of an engine part can be predicted by using a CBM system, which in turn is used to optimally schedule ordering of spare parts and maintenance. We have described a mathematical programming model of this problem for the case of multiple parts, and a dynamic programming algorithm for the special case of a problem with a single part.

Our study showed that the use of a CBM system for the engine parts failure can lead to significant reduction in the delay and improve punctuality. The reduction in cost is highly dependent on the accuracy of the prediction made for the time to failure, which manifests itself in the form of the remaining-life of a part, as well as the lead-time. The implication from the results with a single engine part is that if a failure can be predicted in advance, or a possible failure does not significantly reduce the speed of the ship, it is better to postpone the maintenance or repairs. In contrast, if the remaining-life of a part or the lead-time is short, or if the speed reduction after a failure is significant, then a myopic policy is optimal where maintenance is scheduled at the closest port on the route following the location at which the warning is received. A numerical investigation of the problem with multiple parts revealed that, unless the optimization of the spare parts is done jointly for all parts, then the effectiveness of the CBM system will remain limited, regardless of the quality of the prediction for a single part.

## Footnotes

1 Maximum lead-time seems to be sufficient.
